# Optimal sample size for calibrating DNA methylation age estimators

**DOI:** 10.1111/1755-0998.13437

**Published:** 2021-06-18

**Authors:** Benjamin Mayne, Oliver Berry, Simon Jarman

**Affiliations:** ^1^ Environomics Future Science Platform Indian Ocean Marine Research Centre Commonwealth Scientific and Industrial Research Organisation (CSIRO) Crawley WA Australia; ^2^ School of Biological Sciences The University of Western Australia Perth WA Australia

**Keywords:** age estimation, DNA methylation, epigenetic clock, sample size, wildlife

## Abstract

Age is a fundamental parameter in wildlife management as it is used to determine the risk of extinction, manage invasive species, and regulate sustainable harvest. In a broad variety of vertebrates species, age can be determined by measuring DNA methylation. Animals with known ages are initially required during development, calibration, and validation of these epigenetic clocks. However, wild animals with known ages are frequently difficult to obtain. Here, we perform Monte‐Carlo simulations to determine the optimal sample size required to create an accurate calibration model for age estimation by elastic net regression modelling of cytosine‐phosphate‐guanine methylation data. Our results suggest a minimum calibration population size of 70, but ideally 134 individuals or more for accurate and precise models. We also provide estimates to the extent a model can be extrapolated beyond a distribution of ages that was used during calibration. The findings can assist researchers to better design age estimation models and decide if their model is adequate for determining key population attributes.

## 1 INTRODUCTION

Age is a fundamental parameter in wildlife management. The ages of animals are used to determine population structure, size, the risk of extinction, manage invasive species, and determine the rate of sustainable harvest (Bravington et al., 2016; Bravington et al., 2016; Hoenig, 1983; Tabak et al., 2018). Many wild animals do not exhibit a visible phenotypic age or have a practical and noninvasive method for age estimation. Invasive or opportunistic methods are often used to determine age. For example, in fisheries, counting growth rings in the inner ear bone or the otolith is used to estimate age (Cailliet et al., 2001; Moen et al., 2018). Skeletochronology can also be used to determine age in many vertebrate species, however this can only be carried out on deceased specimens (Guarino et al., 2004; Snover, 2002). Lethal research methods cannot be used on endangered animals and therefore there is a lack of age information in many wild species. Many researchers are turning towards age estimation by DNA methylation as a nonlethal method (De Paoli‐Iseppi et al., 2017; Polanowski et al., 2014). However, the restricted sample sets of known age individuals, which are required to verify age biomarkers, are a key limiting step in applying this approach.

DNA methylation in vertebrates refers to methyl group modification of cytosine‐phosphate‐guanine (CpG) sites and is a common DNA biomarker for age estimation (Field et al., 2018). One of the first studies to develop a model of age was in humans by Horvath, who assembled a data set of 8,000 samples from 51 healthy types of tissues and cell types (Horvath, 2013). The accuracy of the model had a median absolute error (MAE) of 3.6 years. Horvath was able to assemble a large data set by using publicly available databases such as the NCBI Gene Expression Omnibus (Barrett et al., 2012; Horvath, 2013). The human epigenetic clock has also been used to study biological ageing (Petkovich et al., 2017; Salameh et al., 2020). Samples sizes available for wild animals are typically much smaller. Across published studies where successful age models have been developed the minimum sample size is 45 (max = 302 in nonhumans) (Polanowski et al., 2014; Stubbs et al., 2017; Thompson et al., 2017; Wright et al., 2018). Age estimation studies vary in performance and accuracy and one likely contributor to this variability is the sample size.

DNA for methylation analysis can be conducted on single or multiple tissue types. Use of a single tissue source reduces the complexity of the age estimation model (Horvath, 2013; Stubbs et al., 2017). It also reduces the number of CpG sites required and therefore will reduce cost when using technology such as pyrosequencing to measure DNA methylation (Polanowski et al., 2014; Wright et al., 2018). For these reasons, and because collecting multiple tissues may be difficult, in an ecological setting it may be more efficient to focus on age models created from a single readily collected tissue type. The use of some tissue sample types can make epigenetic age estimation nonlethal. The first human epigenetic age estimator used cells collected from human mouth swabs (Bocklandt et al., 2011). A range of other tissue types have been used including whale skin, human blood, ear punches, and bat wing clips (Bell et al., 2019; Little et al., 2020; Polanowski et al., 2014; Wright et al., 2018).

Known age wild animals are rare. The most reliable age estimates involve either tagging or tracking individuals throughout their life. Although some studies have tracked animals since birth, the storage of biological material may be too costly for some research budgets (National Research Council, 2010). Furthermore, long‐lived animals are rarely tracked for enough time to obtain samples from the older individuals. As an extreme example, bowhead whales have had a recorded age of over 200 years, many times that of the career of a research scientist. Obtaining reliably aged samples from the older portion of their population is almost impossible (George et al., 1999). Age estimates for some wild animal species can also be obtained from incremental growth features. Many species accumulate discrete features at a relatively consistent rate. The most widely used are fish otoliths, which accumulate growth rings at a regular rate with increasing age (Das, 1994). Another example is the monthly and daily growth increments of squid statoliths (Rodhouse & Hatfield, 1990). Some species that have nondeterministic growth patterns and change size throughout life can have their age estimated from biometric features with a continuous variation. Examples include fish that follow a von Bertalanffy growth model using age data from otoliths, although we do not know of any epigenetic clocks that have been calibrated from samples with age estimates derived in this manner (von Bertalanffy, 1966; Fabens, 1965).

In biomedical research, an abundance of biological material associated with phenotypic and genomic data is available on online databases. Ecological studies focusing on wild animals do not often have such abundant data. Many ecological studies proceed with low sample sizes for calibration of age models, as they have to make the best with what is available (Di Stefano, 2003; Lemoine et al., 2016). However, age estimation by DNA methylation can be a costly endeavour as it may involve measuring methylation genome wide (Stubbs et al., 2017; Thompson et al., 2017). Studies should ideally pre‐determine whether their sample size is adequate to achieve the accuracy and precision they require for the downstream application.

The lack of known ages in wild animals, especially older individuals in long‐lived species can make it potentially impossible to fully validate an age estimation model. However, it may be possible to provide confidence intervals, error rates, and extrapolate limits given the sample size for any age estimation model. Here, we use a large publicly available human DNA methylation data set as a model to parameterise Monte‐Carlo simulations. We also use a smaller zebrafish (*Danio* *rerio*) data set to show a consistent trend in findings between species. These simulations were used to determine the critical sample size for accurate age estimation. We also determine the effect of different sample age distributions in age estimation. The results provide a guide to the potential accuracy and precision a model is likely to achieve given a sample size.

## 2 MATERIALS AND METHODS

### 2.1 DNA methylation data sets

Although it is possible to generate simulated DNA methylation data, a method that simulates combined methylation and age data does not exist (Rackham et al., 2015). We substituted the lack of simulated data for actual data. The Gene Expression Omnibus, Short Read Archive, and ArrayExpress were searched for the largest DNA methylation data set from one tissue type with associated age information (Barrett et al., 2012; Leinonen et al., 2011; Parkinson et al., 2007). A single tissue type was chosen as multiple tissues can increase the complexity and performance of the model (Stubbs et al., 2017). GSE41037 was identified as the largest data set available and consisted of 394 whole blood samples from healthy human individuals (https://www.ebi.ac.uk/arrayexpress/experiments/E‐GEOD‐41037/) (Wang et al., 2016). We also used nonhuman reduced representation bisulfite sequencing (RRBS) data set consisting of 96 zebrafish caudal fin (https://data.csiro.au/collections/collection/CIcsiro:46344v2/DItrue) (Mayne et al., 2020). The zebrafish data set, despite having a smaller sample size was used to determine if the same trends in the human data set is carried over to a nonhuman data set.

The human data, processed and normalised, was downloaded from ArrayExpress. The zebrafish data was processed using BS‐Seeker2 v 2.0.3 and bowtie2 v2.3.4 using default settings (Guo et al., 2013; Langmead & Salzberg, 2012). Cullen and Frey and quantile‐quantile (Q‐Q) graphs were used to assess the distribution type of the ages in the data set using the R package fitdistrplus (Delignette‐Muller & Dutang, 2015).

### 2.2 Age estimation by DNA methylation

For both the human and zebrafish data we performed Monte‐Carlo simulations to create different sampling scenarios from the complete data sets for age estimation by DNA methylation. The details of specific sample size reductions or distribution iteration are described in the sections below. For each iteration (replicate of a specific sampling scenario), the createDataPartition in the caret R package was used to randomly assign 70% of the samples into a training data set and 30% into a model validating data set and to maintain an equal sex ratio (Kuhn, 2008). The glmnet function in the R package, glmnet was set to a 10‐fold cross validation with the alpha parameter set to 0.5 (Friedman et al., 2010). For each iteration, glmnet selects a set of CpG sites to predict age. Therefore, the total number of CpG sites to predict age is being modified by the model with each iteration. The R code was followed as described by Horvath (refer to Additional File 2 of Horvath, 2013).

### 2.3 Sample size reduction

To determine the minimum sample size for age estimation we performed 100 iterations of the glmnet function for each sample size ranging from 15 to 394 for humans and 15 to 96 for zebrafish with a step size of one. For each iteration an elastic net regression model was performed. The performance of each sample size was determined by the mean Pearson correlation and the MAE rate. The minimum sample size was determined when the correlation approaches the true correlation. The true correlation is when the correlation does not increase with increasing sample size. The corridor of stability (COS), is the width around the true correlation (Schönbrodt & Perugini, 2013). The widths (*w*) of the COS were chosen by following the rules of thumb suggested by Cohen (1992). The three widths (*w* = 0.10, 0.15, 0.20) correspond to a small, medium, and large effect size. The point of stability (POS) is defined when the correlations enter and do not leave the COS and corresponds to the minimum sample size required to achieve the desired accuracy.

### 2.4 Removal of samples by a sliding window

In many wild animal populations, it is difficult to obtain an even distribution of known ages from birth to maximum lifespan. DNA methylation age estimation models for wild animals may be trained on a narrow range of age but the model may be required to extrapolate on a wider range. The aim in this analysis was to determine the performance of a DNA methylation age estimation model on ages that were not present within the training data. A sliding window approach was used to randomly remove samples at varying percentages of the total number of available samples ranging from 10%–90% in intervals of 10% (Datar & Motwani, 2007). Samples were ordered by increasing age and a nominated percentage at intervals of 10% were removed. An elastic net regression model as described in the Age estimation by DNA methylation section of the methods was applied to the remaining samples. A sliding step of one was used for each percentage and the MAE was used to measure the performance of the model for each step. Cohen’s *D* for small, medium, and large effect size (*d* = 0.20, 0.50, 0.80) was used to define the COS from the MAE with 100% of samples (Cohen, 1992). Only the human data set was used for the sliding window analysis as it had a large sample size to make multiple reductions of samples.

## 3 RESULTS

### 3.1 DNA methylation data set

The human DNA methylation data set consisted of 394 samples, a sex ratio of 192:202 (female: male), and ages ranged from 16–88 years. The data set contained 26,486 CpG sites after probes that mapped to the sex chromosomes were removed. The ages fitted a uniform distribution as shown by the Cullen and Frey and Q‐Q plots (Figure S1A–D). This made it an ideal substitute data set for simulation data as the ranges of age was uniformly distributed (Figure S1A).

The zebrafish data set contained 96 samples with an equal sex ratio. The data set contained ages from 11.9 to 60.1 weeks and a total of 524,038 CpG sites. There was no removal of sex‐specific CpG sites as zebrafish do not have sex chromosomes (Sola & Gornung, 2001; Wallace & Wallace, 2003). The ages of zebrafish were uniformly distributed making it also an ideal substitute for simulation data (Figure S2A–D).

### 3.2 Minimum sample size for age estimation by DNA methylation

**FIGURE 1:**
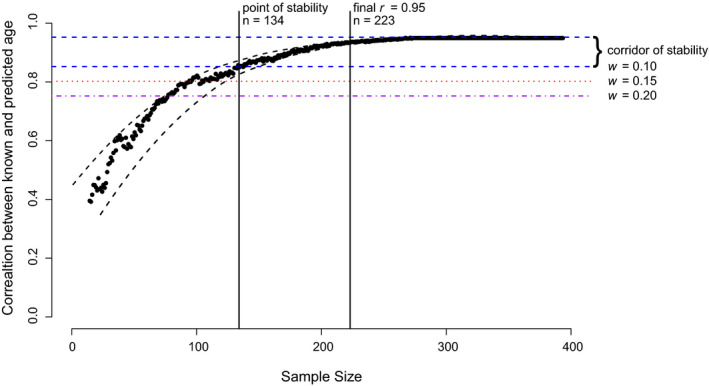
Approaching mean correlations towards the true correlation between the chronological and predicted age with increasing sample size. Each black dot represents the mean correlation between the chronological and predicted age from 100 iterations. Black dashed lines represent the 95% confidence interval range of the mean correlations. Horizontal coloured lines show the corridor of stability at the three different widths

In the human data set the average Pearson correlation between the chronological known and predicted age in the testing data set was used to determine the POS. The first POS at the largest effect size, *w* = 0.20 and at 80% level of confidence was reached at 70 samples (Table 1). The POS with the smallest effect size, *w* = 0.10 and within the 95% percentile was reached with 134 samples (Figure 1). The final most accurate correlation (*r* = .95) was achieved at 223 samples.

**TABLE 1:** Sample sizes at different points of stability, widths, and percentiles for accurate age estimation in both the human and zebrafish data set

Width (*w*)	Percentile	Sample size (human data set)	Sample size (zebrafish data set)^a^
0.10	80%	128	61
0.15		88	57
0.20		70	51
0.10	90%	130	62
0.15		90	58
0.20		76	52
0.10	95%	134	66
0.15		100	58
0.20		78	53

^a^ The zebra fish data is included for comparison only as there were not enough samples available to fully inform any conclusions on calibration population sample size available.

It is important to note the zebrafish data set with only 96 samples can only be used as a guide analogous to human data set. The zebrafish data set reached a POS (*w* = 0.10 and within the 95% percentile) at 66 samples (Table 1 and Figure S3). At 91 samples the Pearson correlation between known and predicted age is increasing, as opposed to plateauing at this sample size in the human data set. This reinforces the value of the human data set which contains 394 samples in this study as it has an excess of samples to draw statistical conclusions. The increasing correlations in the zebrafish data set suggests the data set is not large enough to conclusively identify the optimal sample size. However, the data set shows that an increase in sample size produces better performing models. The zebrafish data set is most likely returning an underestimate of the adequate sample size for age estimation.

### 3.3 Increasing number of CpG predictors with sample size

For each iteration, the number of CpG sites selected by the model to predict age was found to increase with sample size (Figure S4). The highest mean CpG sites for age estimation was 167 in the human data set and 78 in the zebrafish data set. This difference accords with the larger range of sample sizes testable with the human data (*n* = 394) than the zebrafish data (*n* = 96). As described above the zebrafish data set does not contain sufficient samples to use as a definitive guide. It does show a consistent trend in sample size and CpG sites required for age estimation.

### 3.4 Sample distribution and extrapolating the model beyond trained range

**FIGURE 2:**
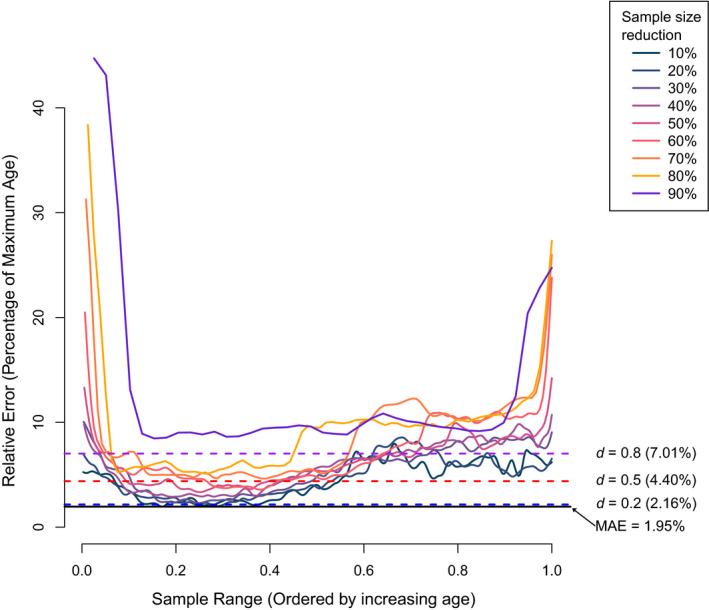
Increasing relative error, as a percentage of the oldest individual in the dataset with increasing removal of sample sizes. Coloured lines show the trajectory of the relative error as the sliding scale with a step of 1 moves along the samples arranged from youngest to the oldest. Horizontal coloured dashed lines show the Cohen's *D* effect sizes

Sample size reduction had the largest effect on relative error when samples were removed from the outer sample range (Figure 2). For each percentage the range had a sliding step of one sample, which provides an indication of how well an age estimation model can be extrapolated. Not surprisingly, the removal of 10% of the samples had the largest range within the smallest effect size (*d* = 0.20), between the first 16.3%–37.5% of the age range (Table 2). Another interpretation of the sliding window analysis is it provides ranges and effect sizes to the extent that the error rate will increase if there is a lack of training data. For example, using Table 2, if we consider the removal of 30% of samples, we find that this increases the relative error beyond the smallest effect size (*d* = 0.20). We then find between the first 10.5%–48.6% age range that the removal of 30% of the samples does not exceed the medium effect size from the true relative error (*d* = 0.50). Between the first 4.0%–73.6% age range, the removal of 30% of samples does not exceed the largest effect size (*d* = 0.80). Table 2 can serve as a guide for researchers to decide if it is worth inputting the time and resources in developing an age estimation model with the known age animals that are available.

**TABLE 2:** Point of stabilities at increasing widths of effect size (*d*) for the human data. Point of stabilities are defined in terms of the percentage of samples arranged from the youngest to oldest individual. NA refer to not available as the relative error exceeded the effect size

	*d* = 0.20		*d* = 0.50		*d* = 0.80	
Sample size reduction (%)	Minimum sample (%)	Maximum sample (%)	Minimum sample (%)	Maximum sample (%)	Minimum sample (%)	Maximum sample (%)
10	16.3	37.5	7.0	54.6	0.3	100
20	24.1	29.8	6.3	53.0	0.3	100
30	NA	NA	10.5	48.6	4.0	73.6
40	NA	NA	8.5	42.4	3.4	67.8
50	NA	NA	9.6	39.6	6.1	70.6
60	NA	NA	26.6	41.1	5.1	63.9
70	NA	NA	NA	NA	5.9	55.9
80	NA	NA	NA	NA	6.3	44.3
90	NA	NA	NA	NA	NA	NA

## 4 DISCUSSION

Age estimation of animals from DNA methylation is a useful method that can help monitor population sizes and inform predictions of their growth rate. Using DNA methylation for wild animal age estimation and subsequent application in studies of population biology is a relatively new field of research. Age estimation studies by DNA methylation in wild animals vary greatly and no framework currently exists to facilitate better best practises for model development. One of the fundamental aspects of developing a model for age estimation is the calibration population sample size, but it is rarely mentioned in the literature. Although it is fully acknowledged that known age wild animals are rare for a multitude of reasons and most studies can only work with what is available.

Developing a DNA methylation age estimation model requires animals of known ages to calibrate and validate the model. The lack of known ages in wild animals can result in underperforming models that are not adequate for downstream analyses. Our simulations based on a large human data set demonstrated the importance of adequate sample size and that the minimum required was 70 and ideally should approach 134 (Figure 1). The zebrafish results also show a similar trend but due to the smaller size of the data set it is difficult to draw any definitive conclusions. It is most likely that the zebrafish data set provides an underestimate of the required sample sizes. Although larger data sets do exist in other nonhuman species such as mice, these are from multiple laboratories, tissue types, and sequencing batches increasing the complexity of the data set (Stubbs et al., 2017). The human data set used in this study is ideal as it a single tissue type and not from multiple laboratories or batches, thereby reducing any external influences.

In the literature the smallest sample size used for an age estimation model is 45 in Humpback whales (Polanowski et al., 2014). A statistically significant *R* ^2^ of .787 was reported between the known and predicted age in humpback whales (Polanowski et al., 2014). An epigenetic age estimator in Bechstein’s bats produced a *R* ^2^ of .58 but used 62 samples (Wright et al., 2018). The humpback whale and Bechstein’s bat studies demonstrate low samples sizes can still produce accurate models. However, they also demonstrate the variation between model performance with sample sizes <70. This recommendation is not meant to be critical of these studies specifically, since known age wild animals are in short supply and often researchers will have to make the best with what is available. However, our results are valuable for the planning of future age estimation studies. A key component of planning this sort of research is the required precision and accuracy for an age estimator. This will depend on the intended use of the data, so likely error magnitude should be considered when calibration sample sets are small. Alternative age estimation methods may be available, or if no practical alternatives exist then even models that deliver high error may still provide valuable information for species management. For example, in many fisheries, highly accurate models are often required as age is used for to determine the sustainable harvest (Beamish & McFarlane, 1983; Campana, 2001). In contrast, when determining population age structure and mortality rates, some level of inaccuracy and bias can be tolerated so long as animals can be easily placed into age classes (Caughley, 1966, 1977). For close kin mark recapture estimates of population size, the order of age in kin pairs improves the estimate. Determining age order is not dependent on accuracy, but is affected by precision (Bravington et al., 2014; Polanowski et al., 2014). Our analyses provide a framework to determine if future age estimation studies are likely to be adequately statistically powered.

Another important factor to consider when developing age estimation models is the number of predictors or CpG sites included in the model since this has implications for how they can be assayed routinely. In both the human and zebrafish data the optimal number of CpG sites per model increased with increasing sample size. The optimal number of CpG plateaued against samples in both data sets. Interestingly, the number of CpG sites at the same sample size differs between data sets. For example, the mean number of CpG sites in models based on 96 samples was 22 for the human data set and 78 for the zebrafish. The differences may reflect differences between tissue types. Whole blood was used for the human data set, whereas caudal fin tissue was used for the zebrafish. Caudal fin tissue may be more heterogenous than whole blood and therefore requires a higher number of CpG sites to accurately predict age. The number of CpG sites used for age estimation range from three to 353 CpG sites in nonreproductive tissue (Horvath, 2013; Polanowski et al., 2014). The mouse (329 CpGs) and human (353 CpGs) age estimation models have the most CpG in the literature and are both multitissue models. In human placental tissue, two epigenetic clocks have been developed. The first was developed with 170 samples and uses 62 CpG sites, and the second with 1,102 samples and 558 CpG sites (Lee et al., 2019; Mayne et al., 2017). One suggested possibility for the difference in number of CpG sites selected for each of the two epigenetic clocks is biological effects (Lee et al., 2019). The epigenetic clock with the larger sample size may have captured more biological variation and requires more CpG sites to accurately predict age. The number of CpG sites a model requires to accurately estimate age may be more linked to an array of biological effects including but not limited to, sample size, tissue type, the number of tissues and organs, and heterogeneity.

The range of ages in a calibration data set can also influence the performance of an age estimation model. In wildlife research if known ages are available, they would most likely be skewed towards younger individuals, which are typically more common (Müller et al., 2004). Consequently, age estimation models developed from data dominated by younger individuals may unintentionally perform better for younger than older individuals. The removal of samples in the sliding window analysis shows that the relative error can increase with an effect size of 0.80 with removal of only 10% of samples from any position with the range of age (Table 2). This suggests a uniform age distribution is ideal and confirms that the performance is reduced for models derived from skewed age distributions. The models were more sensitive to missing data at the upper and lower ranges (top and bottom 10%) than to the middle 80% (Figure 2 and Table 2). For example, the model developed from data missing 50% of the individuals between the 9.6%–39.6% of the full age range, will not have effect size greater than *d* = 0.50. In other words, the relative error of this model will not exceed 125% of the error from the model with all the samples (MAE from 1.95% to 4.40%). On the other hand, a model developed from data missing 50% of the individuals but was outside of the 9.6%–39.6% of the full age range will increase the relative error 260% above the original model with all the samples (MAE > 7.01%). The sliding window analysis suggests when an age series has missing data the model will perform better by extrapolating inwards with known data than on the outer limits of the age distribution.

The lack of known age wild animal samples can be corrected for some species by developing an epigenetic clock on laboratory specimens. This can make multiple individuals of the same age available, allowing for more robust initial models and providing the opportunity to independently validate the clock on a different known age sample set. Laboratory‐reared specimens can be straightforward to obtain for short‐lived species such as the zebra fish analysed here. Long‐lived species are more challenging, and many cannot be kept in captivity, but these are the species where age estimation is most valuable for their population management. It may be desirable to validate the model on closely related, short‐lived species and then apply the markers to the longer‐lived species. It appears that many CpG sites have age‐related DNA methylation in near relatives of that species (Beal et al., 2019; Horvath, 2013; Polanowski et al., 2014).

Age estimation is a fundamental resource for wildlife management in a wide variety of applications. Due to the limited resource of wild animals with known ages studies, we must inevitably develop models from whatever samples are available. This has the potential to result in statistically underpowered models. A consequence of low sample sizes may result in inaccurate age estimation models. By performing Monte‐Carlo simulations we have shown that as a rule of thumb the minimum sample size to develop a model with good accuracy and precision is 70 individuals and ideally it should approach 134. Furthermore, wherever possible samples should have as even a distribution across the range of ages, and ideally include animals form within the top and bottom 10% of known ages.

## AUTHOR CONTRIBUTIONS

Benjamin Mayne conceived, designed, carried out the study, analysed and interpreted the data and wrote the manuscript. Oliver Berry and Simon Jarman provided discussion and intellectual input into the manuscript. All authors read and approved the final version of the manuscript.

## Supporting information

Fig S1

Fig S2

Fig S3

Fig S4

Supplementary Material

## Data Availability

Data sharing is not applicable to this article as no new data were created or analysed in this study.
