# Supplementary figures and images for: Optimal sample size for calibrating DNA methylation age estimators
Source: Mol Ecol Resour. 2021 Jun 18;21(7):2316–23. doi: 10.1111/1755-0998.13437 (PMC8518423; doi:10.1111/1755-0998.13437)

**A**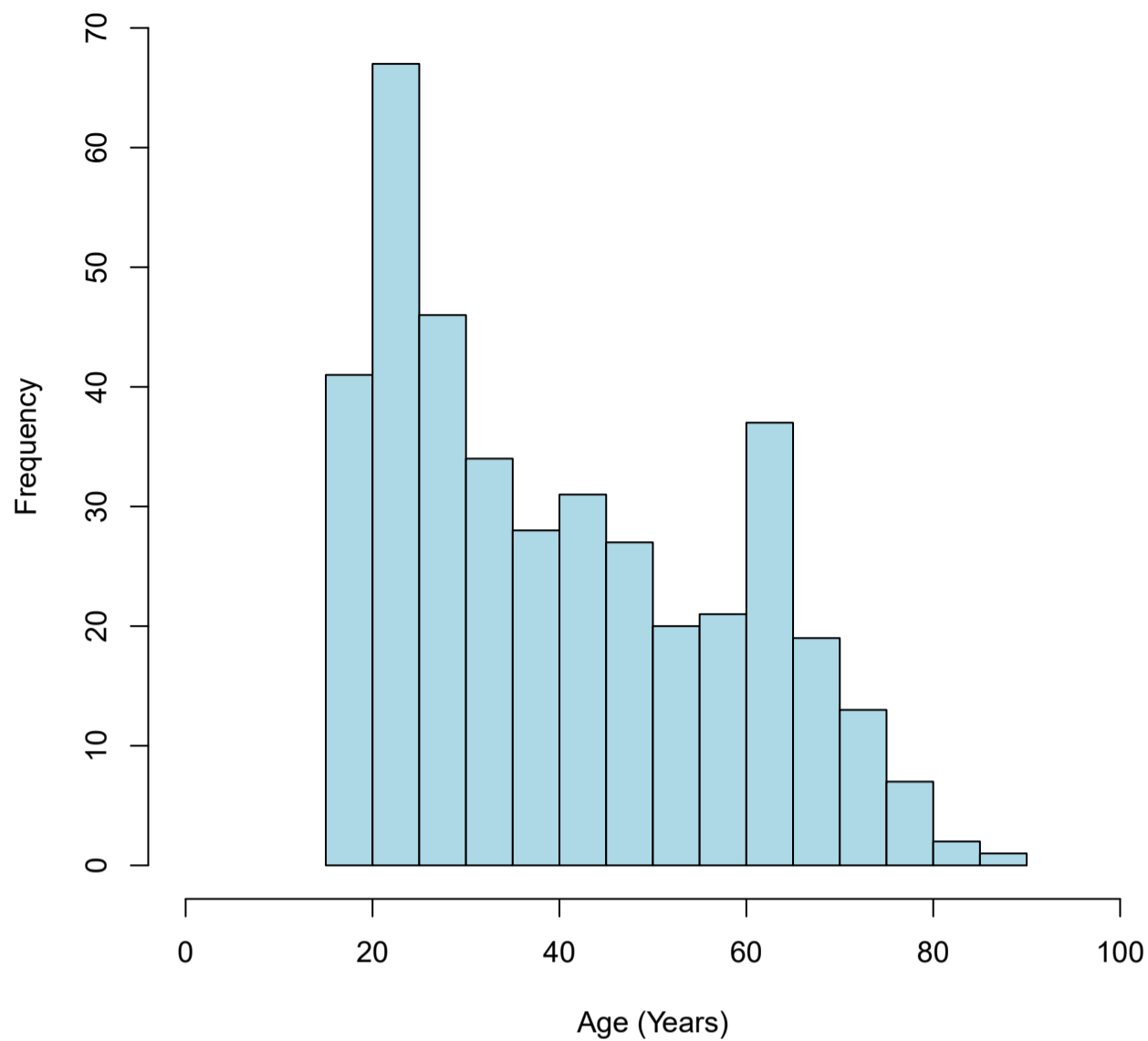**C**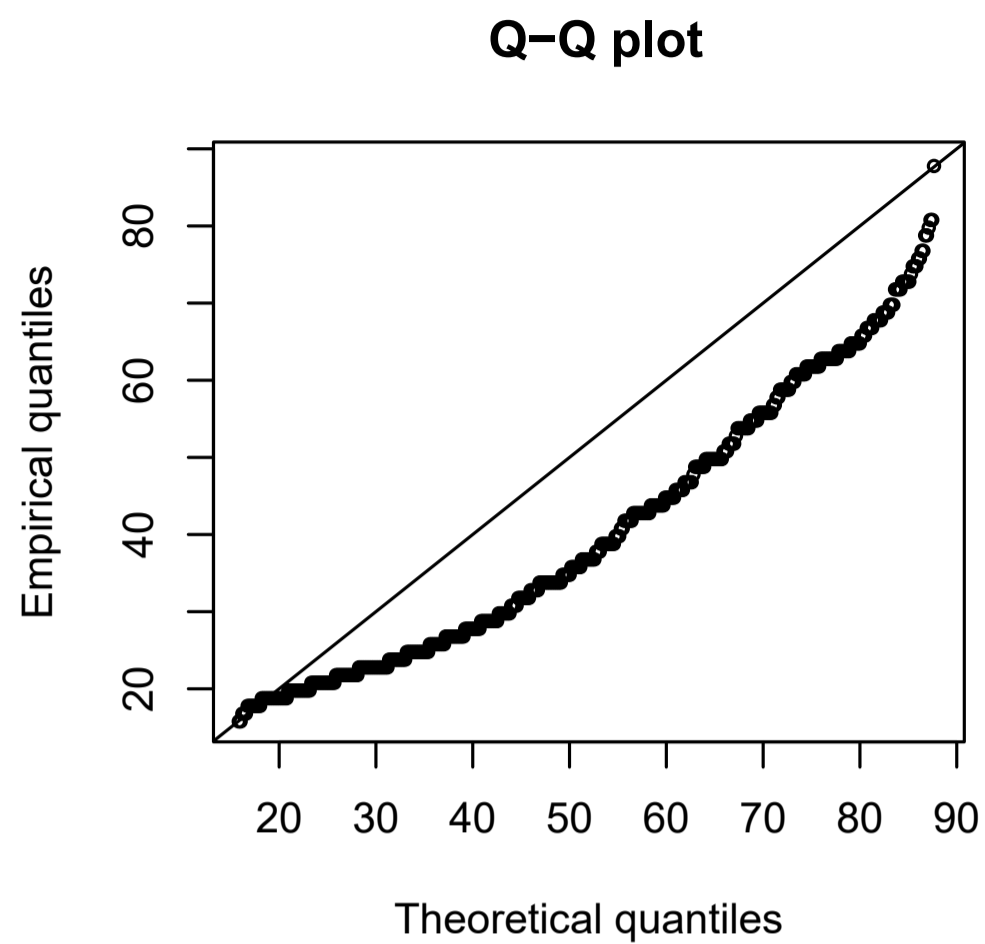**B****Cullen and Frey graph**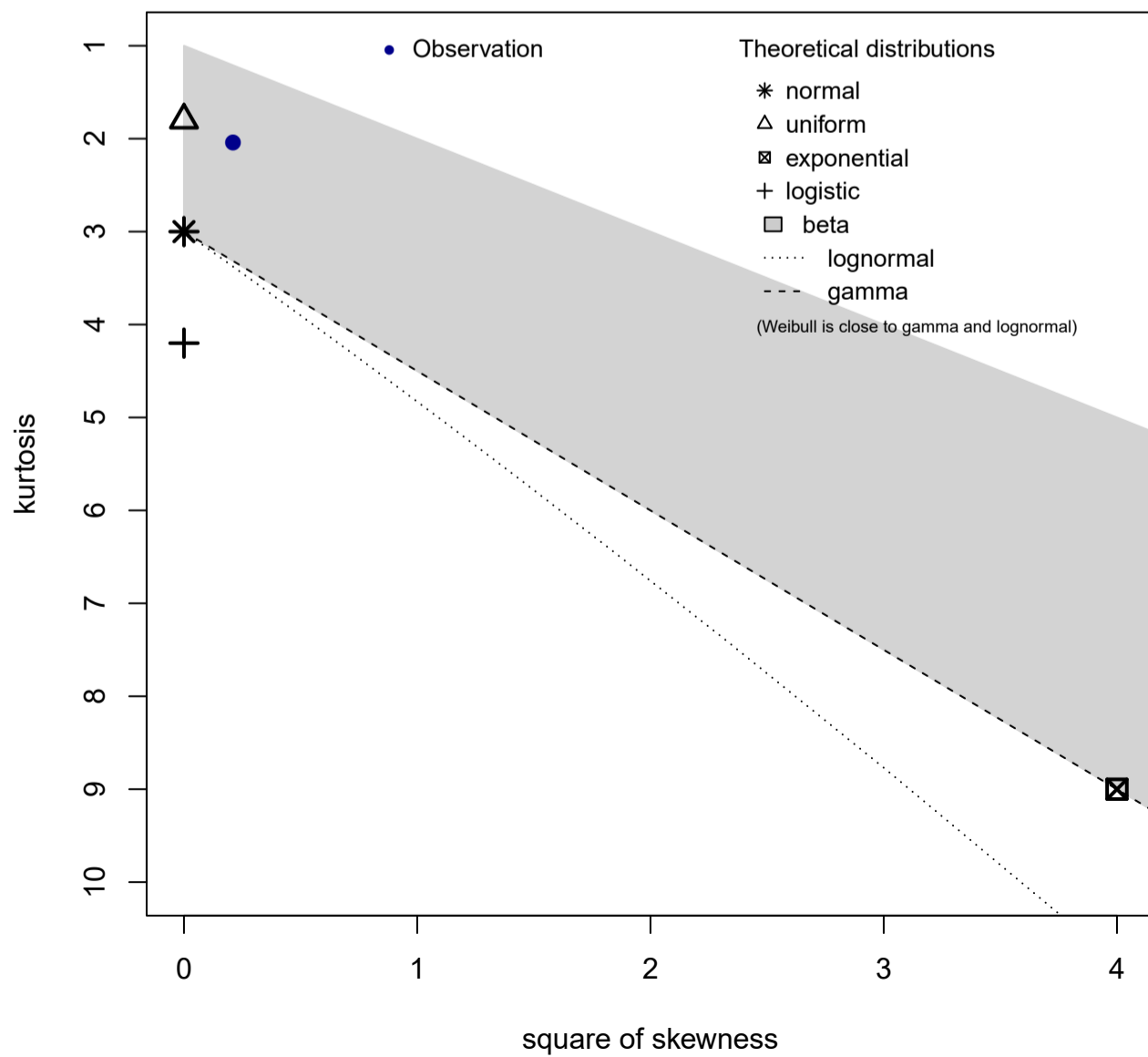**D**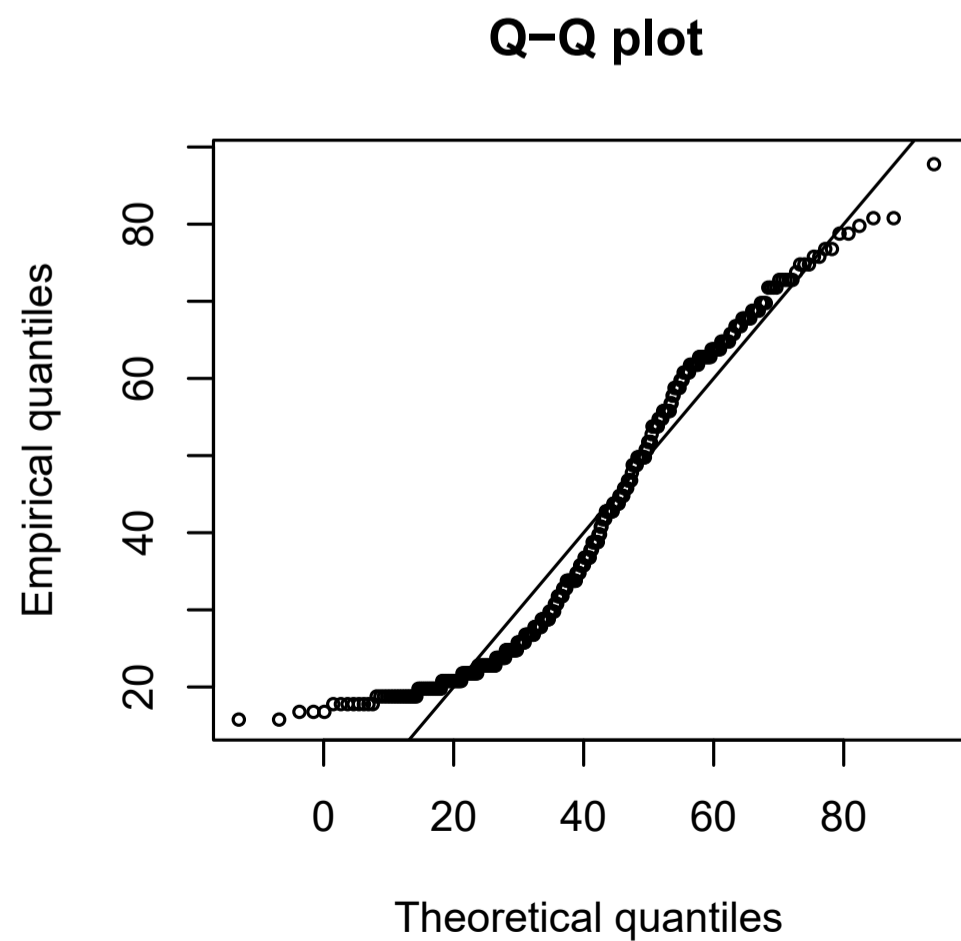

Supplement: Supplementary file 1 — Fig S1 [file MEN-21-2316-s002.pdf]

**A**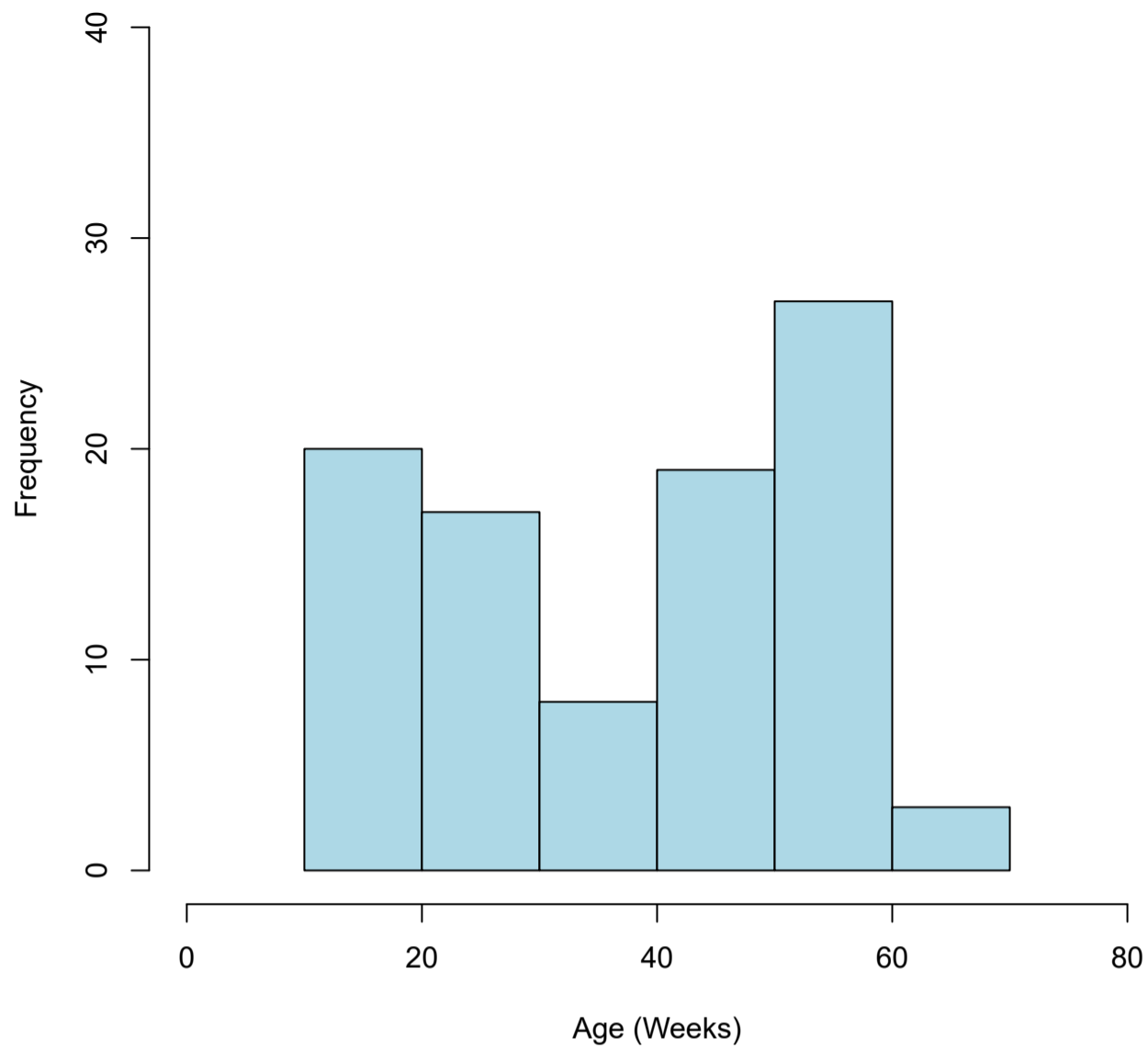**C**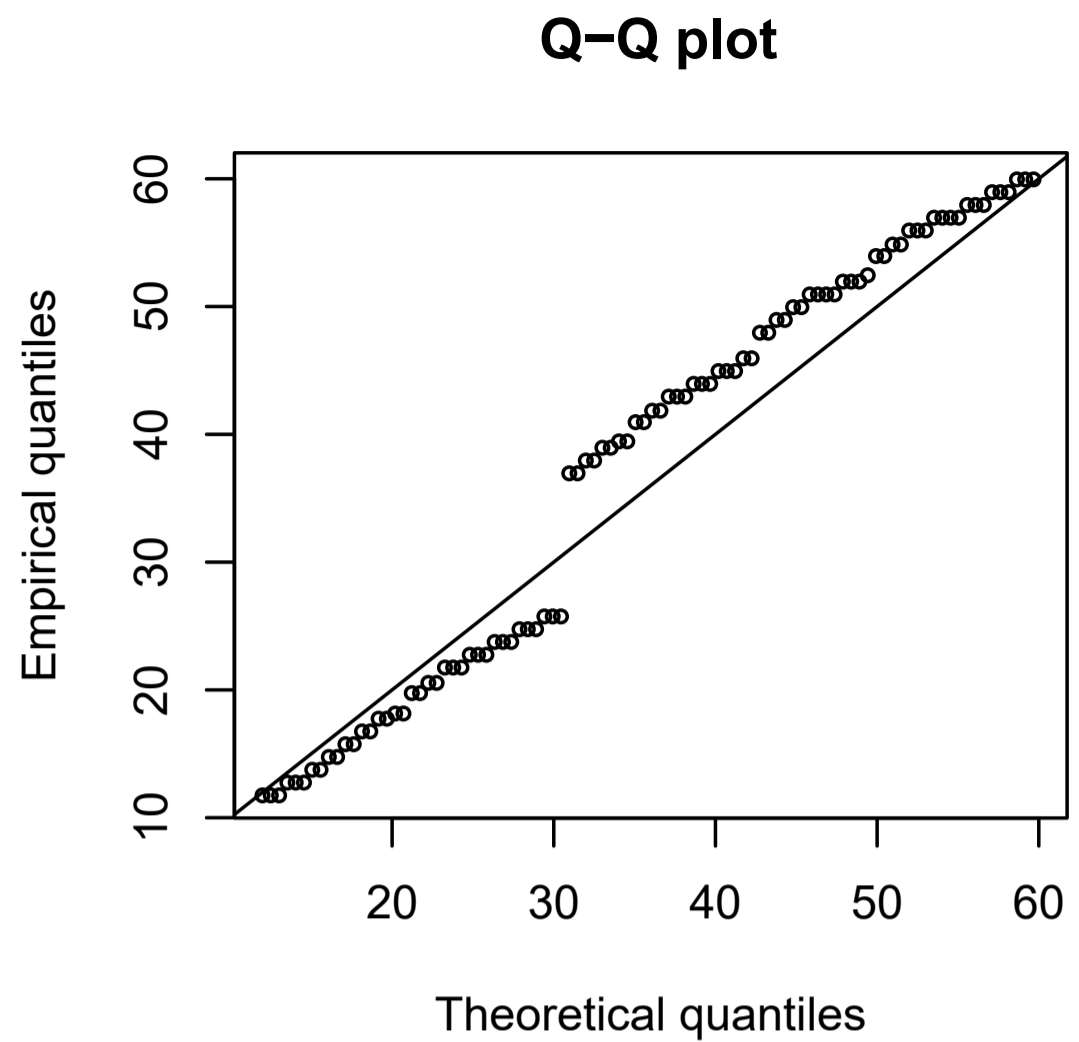**B**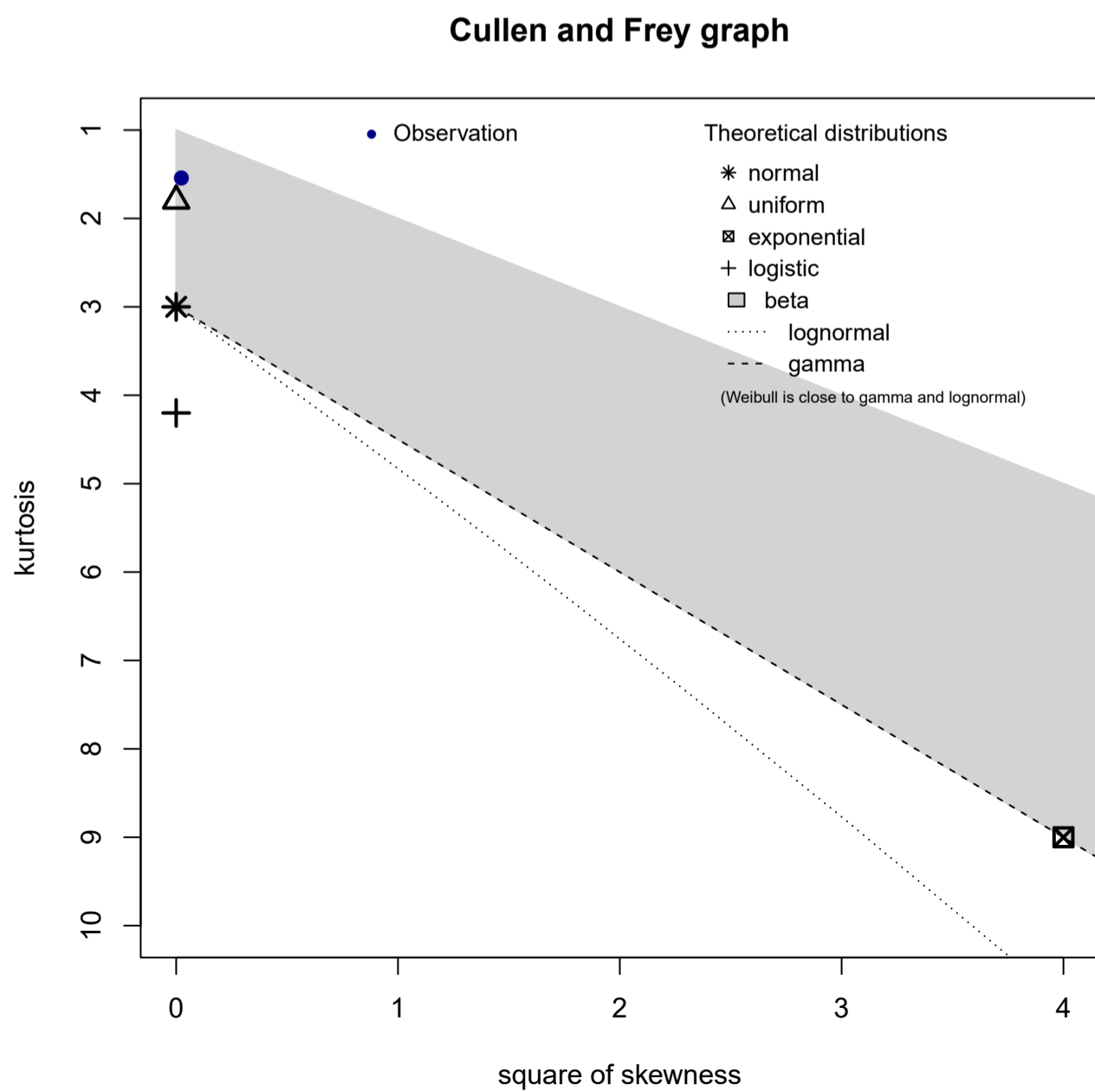**D**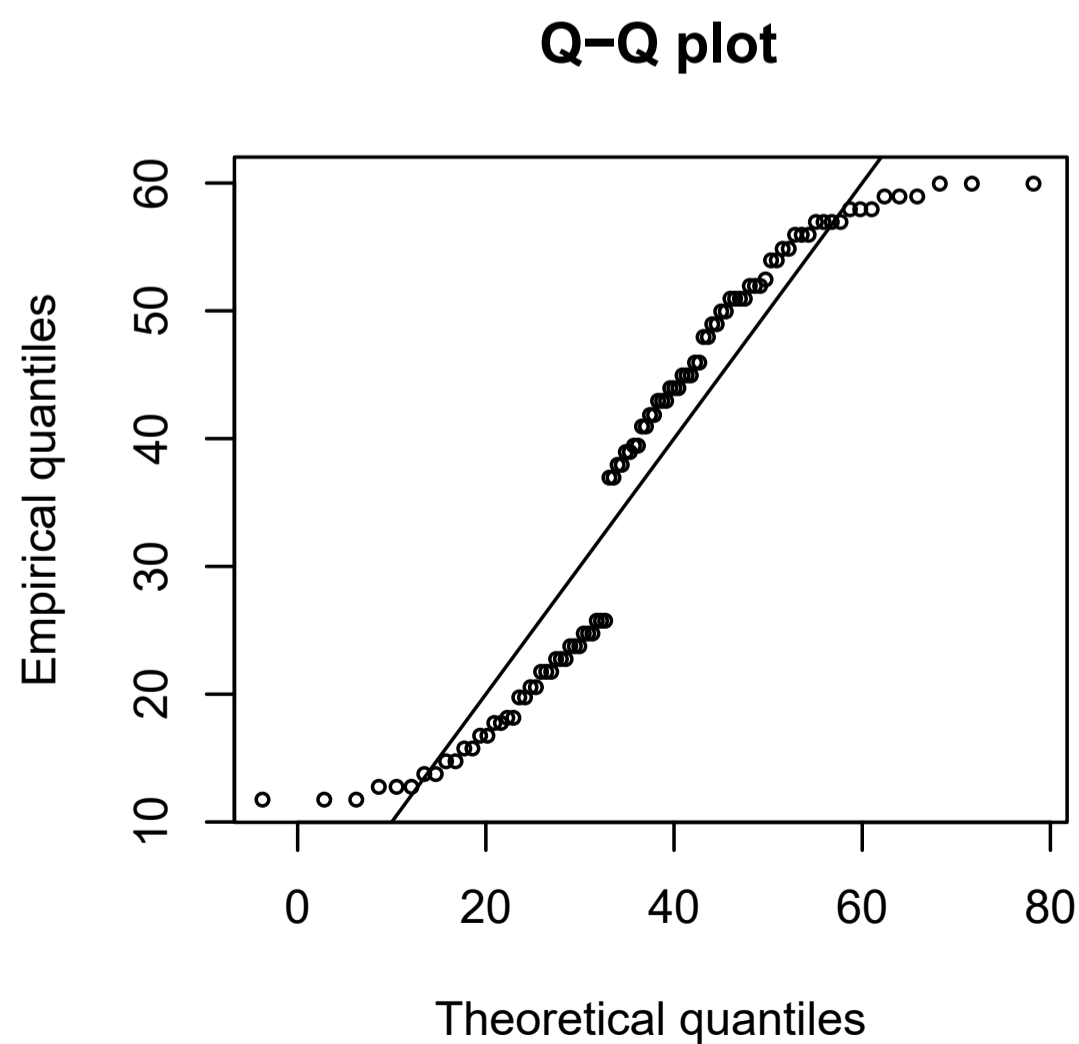

Supplement: Supplementary file 2 — Fig S2 [file MEN-21-2316-s004.pdf]

Correlation between known and predicted age

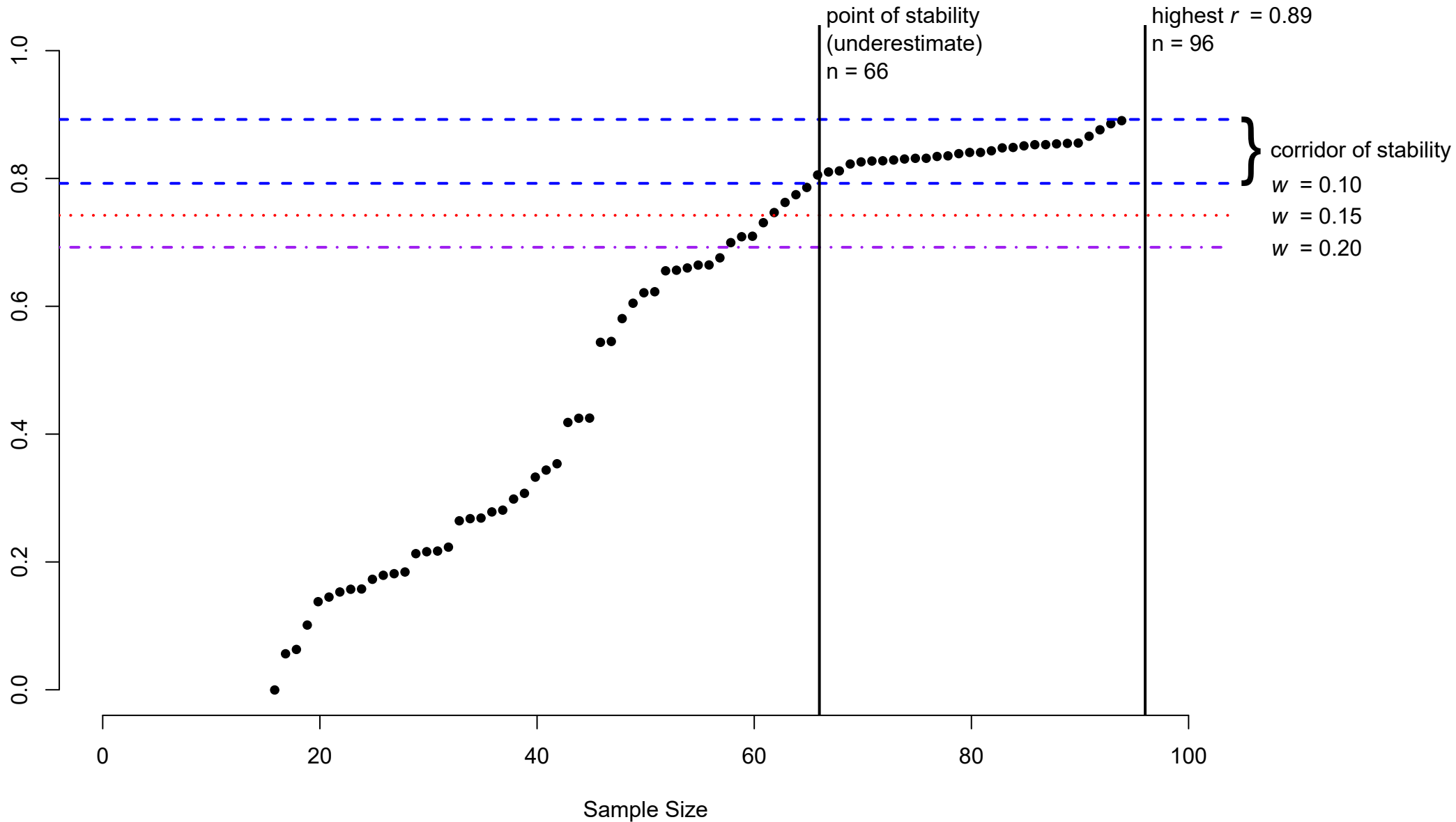

Supplement: Supplementary file 3 — Fig S3 [file MEN-21-2316-s003.pdf]

A

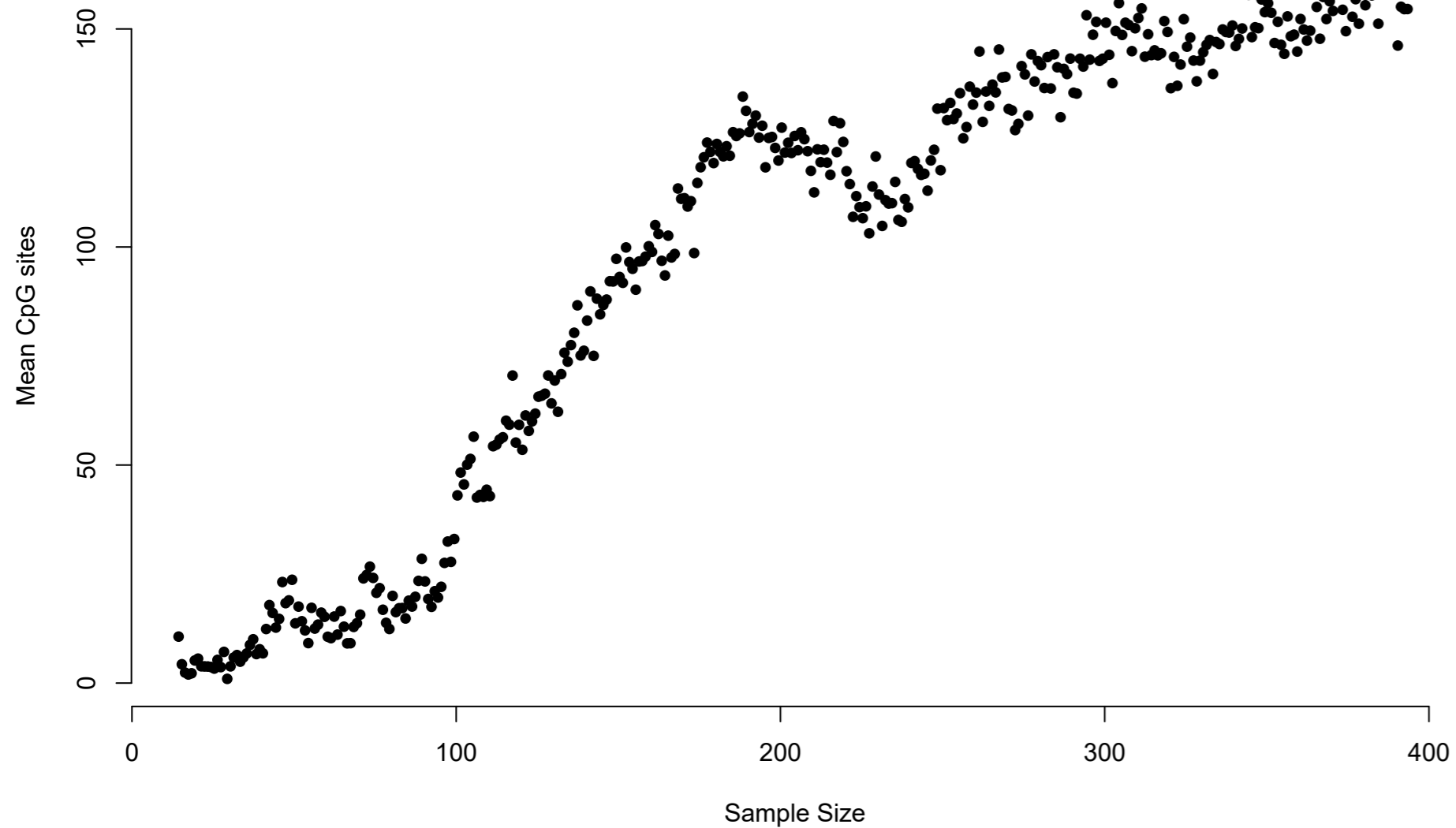

B

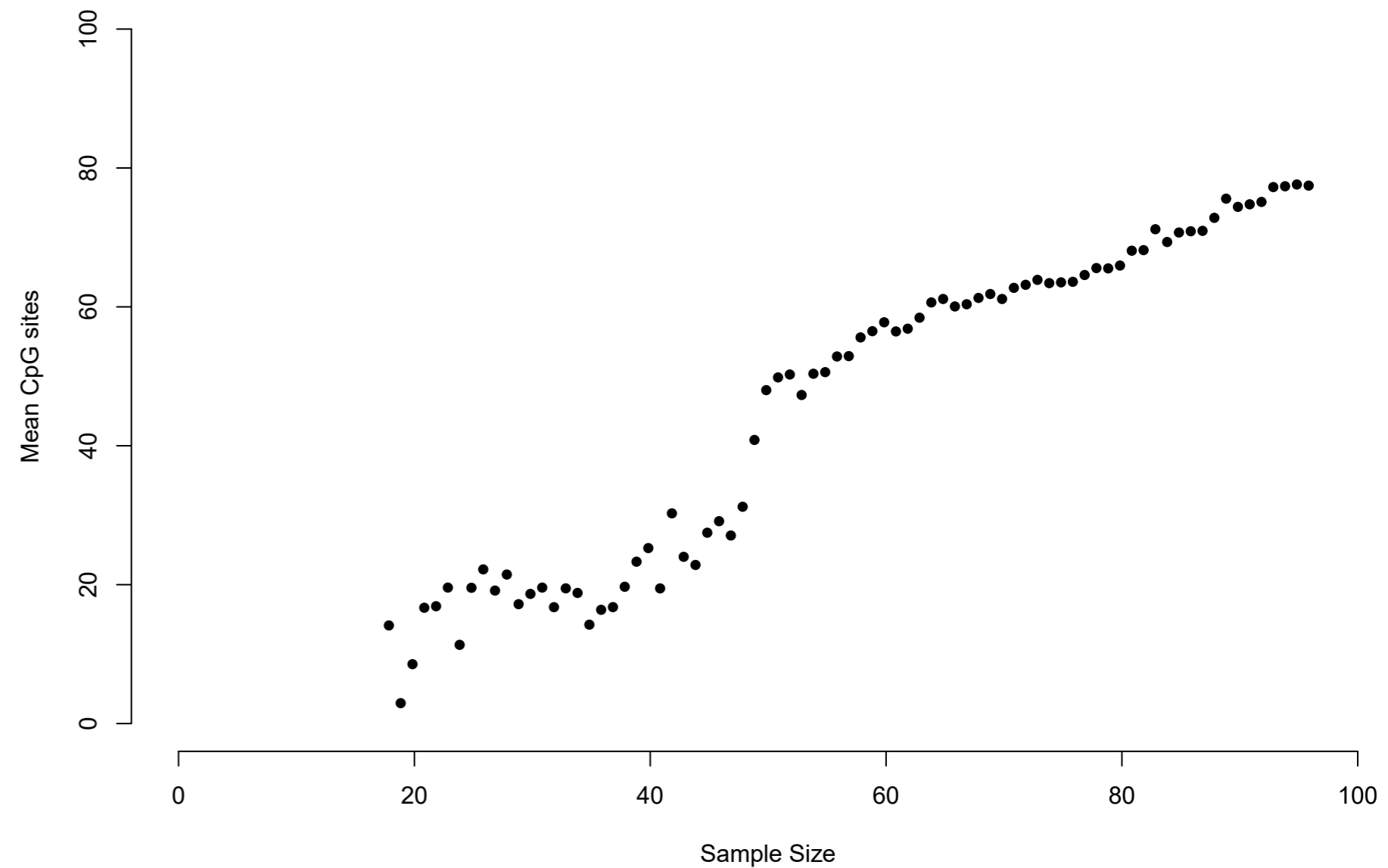

Supplement: Supplementary file 4 — Fig S4 [file MEN-21-2316-s001.pdf]
